# COVID-19 and Skin Manifestations: An Overview of Case Reports/Case Series and Meta-Analysis of Prevalence Studies

**DOI:** 10.3389/fmed.2020.573188

**Published:** 2020-10-29

**Authors:** Fatemeh Sameni, Bahareh Hajikhani, Somayeh Yaslianifard, Mehdi Goudarzi, Parviz Owlia, Mohammad Javad Nasiri, Shervin Shokouhi, Mahmood Bakhtiyari, Masoud Dadashi

**Affiliations:** ^1^Department of Microbiology, Faculty of Medicine, Shahed University, Tehran, Iran; ^2^Department of Microbiology, School of Medicine, Shahid Beheshti University of Medical Sciences, Tehran, Iran; ^3^Department of Microbiology, School of Medicine, Alborz University of Medical Sciences, Karaj, Iran; ^4^Molecular Microbiology Research Center, Shahed University, Tehran, Iran; ^5^Infectious Diseases and Tropical Medicine Research Center, Shahid Beheshti University of Medical Sciences, Tehran, Iran; ^6^Department of Infectious Diseases and Tropical Medicine, Loghman Hakim Hospital, Shahid Beheshti University of Medical Sciences, Tehran, Iran; ^7^Non-communicable Diseases Research Center, Alborz University of Medical Sciences, Karaj, Iran; ^8^Department of Community Medicine, Alborz University of Medical Sciences, Karaj, Iran

**Keywords:** coronavirus, 2019-nCoV, COVID-19, skin manifestations, meta-analysis

## Abstract

**Background and Aim:** Since the onset of the 2019-nCoV disease (COVID-19), many skin manifestations have been reported in COVID-19 patients. This study aims to provide a systematic review and meta-analysis of various skin manifestations among patients with COVID-19 through case reports/case series and prevalence studies.

**Methods:** A systematic literature search strategy was conducted by reviewing original research articles published in Medline, Web of Science, and Embase databases in 2020. Statistical analysis was performed using STATA software, version 14.0 (Stata Corporation, College Station, Texas, USA) to report the global prevalence of skin manifestations among patients with COVID-19.

**Results:** Forty-three studies (35 articles were case reports/case series, and 8 articles were prevalence studies) were included in our study. A meta-analysis of prevalence studies showed that skin manifestations among patients with COVID-19 were reported in four countries (China, Thailand, France, and Italy), with an overall prevalence of 1.0% [(95% CI) 0.1–1.9] among 2,621 patients. Evaluation of the results of the case reports/case series revealed that, out of 54 patients with COVID-19, 48 patients (88.8%) showed skin manifestations. Erythematous rash (59.1%) and urticaria (14.8%) were the most common skin manifestation reported in studied patients.

**Conclusion:** Infection with 2019-nCoV may lead to skin manifestations with various clinical symptoms. These clinical features combined with clinical symptoms of COVID-19 may aid in the timely diagnosis of patients with COVID-19.

## Introduction

The first reports of people infected with 2019 novel coronavirus (2019-nCoV) were published in December 2019 in Wuhan, China. Afterwards, the pandemic spread rapidly across the world (1–3). The symptoms of 2019-nCoV disease (COVID-19) vary from person to person, and it covers a wide range of clinical manifestations (4). However, most of the patients presented mild or no symptoms (5–7). Older people and those who suffer from underlying medical conditions such as high blood pressure, heart disease, or diabetes seem to be at higher risk for developing more serious complications of COVID-19 (8). According to studies, the most common symptoms of COVID-19 are fever, tiredness, and dry cough. Also, some patients may experience muscle pains, runny or stuffy nose, sore throat, gastrointestinal symptoms, and loss of smell and taste (9–11). In addition to these symptoms and according to the results of studies on COVID-19 patients, different types of skin manifestations have been seen in a number of patients (12, 13). The skin involvement in patients with COVID-19 was not noticed at the early stages of this pandemic, but it has received much more attention recently (14). The most important skin manifestations in people with COVID-19 are red spots on the hands, blisters on the trunk, and itchy hives (14). In some COVID-19 patients, red patches of itchy skin, associated with skin inflammation, have been observed as well. These lesions affect the hands and feet and may look like small, swollen, itchy blisters (15). Despite the observation of skin manifestations in patients with COVID-19, researchers are still looking for answers to the question of whether this skin presentations are directly related to the virus itself or are complications of the infection (16). In addition, in many cases, skin problems in COVID-19 patients may be due to drug side effects and the virus may not be the cause (17, 18). Therefore, it seems that finding out the potential relationship between COVID-19 and skin manifestations can assist in better understanding the pathogenesis of the disease and adoption of better infection control policies. The present study aimed to investigate the distribution, types, and the most prevalent skin manifestations among patients with COVID-19 based on case reports/case series and prevalence studies around the world.

## Methods

### Search Strategy

A broad systematic literature review was conducted via reviewing original research articles published in Medline, Web of Science, and Embase databases in 2020. The following phrases were used in the search strategy of this article: COVID OR COVID-19 OR novel coronavirus OR new coronavirus OR coronavirus 2019 OR 2019-nCoV OR nCoV OR CoV-2 OR SARS-2 OR SARS-CoV-2 OR severe acute respiratory syndrome coronavirus 2 OR skin manifestation OR cutaneous manifestation OR urticaria OR exanthem OR rash OR livedo reticularis OR eczema OR skin OR cutaneous OR skin disease OR dermal disease OR skin lesion. In order to identify further studies, bibliographies of related articles were also screened.

### Inclusion and Exclusion Criteria

We evaluated all case reports/case series and prevalence studies that were about the prevalence of skin manifestations among patients with COVID-19. These studies reported sufficient data for analysis, including country of origin, the number of patients with COVID-19, number of patients with skin manifestations, type of skin manifestations, clinical symptoms, laboratory findings, outcomes, diagnostic methods, and treatment. Titles, abstracts, and full texts of the recorded studies were evaluated based on the inclusion and exclusion criteria. The exclusion criteria were as follows: (1) animal research only, (2) studies considering skin manifestation only, (3) studies considering patients with COVID-19 only, (4) review articles, (5) abstracts presented in conferences, and (6) duplicate studies. Appropriate papers were selected by BH and MG after evaluating all studies based on the inclusion and exclusion criteria.

### Data Extraction and Definitions

The following items were considered in each study: the last name of the first author, study time, time of publication, country, number of patients with COVID-19, number of patients with skin manifestations, type of skin manifestations, clinical symptoms, laboratory findings, outcomes, diagnostic methods, and treatment. Two independent individuals collected the data and another researcher confirmed them.

### Meta-Analysis

Statistical analysis was performed using STATA software, version 14.0 (Stata Corporation, College Station, Texas, USA) to report the global prevalence of skin manifestations among patients with COVID-19. The fixed-effects (FEM) (19) and the random-effects models (REM) were utilized for data collection. Statistical heterogeneity was assessed using the *Q*-test and the *I*^2^ statistical methods. *P* < 0.05 was regarded as statistically significant.

## Results

### Characteristics of Included Studies

**Figure 1:**
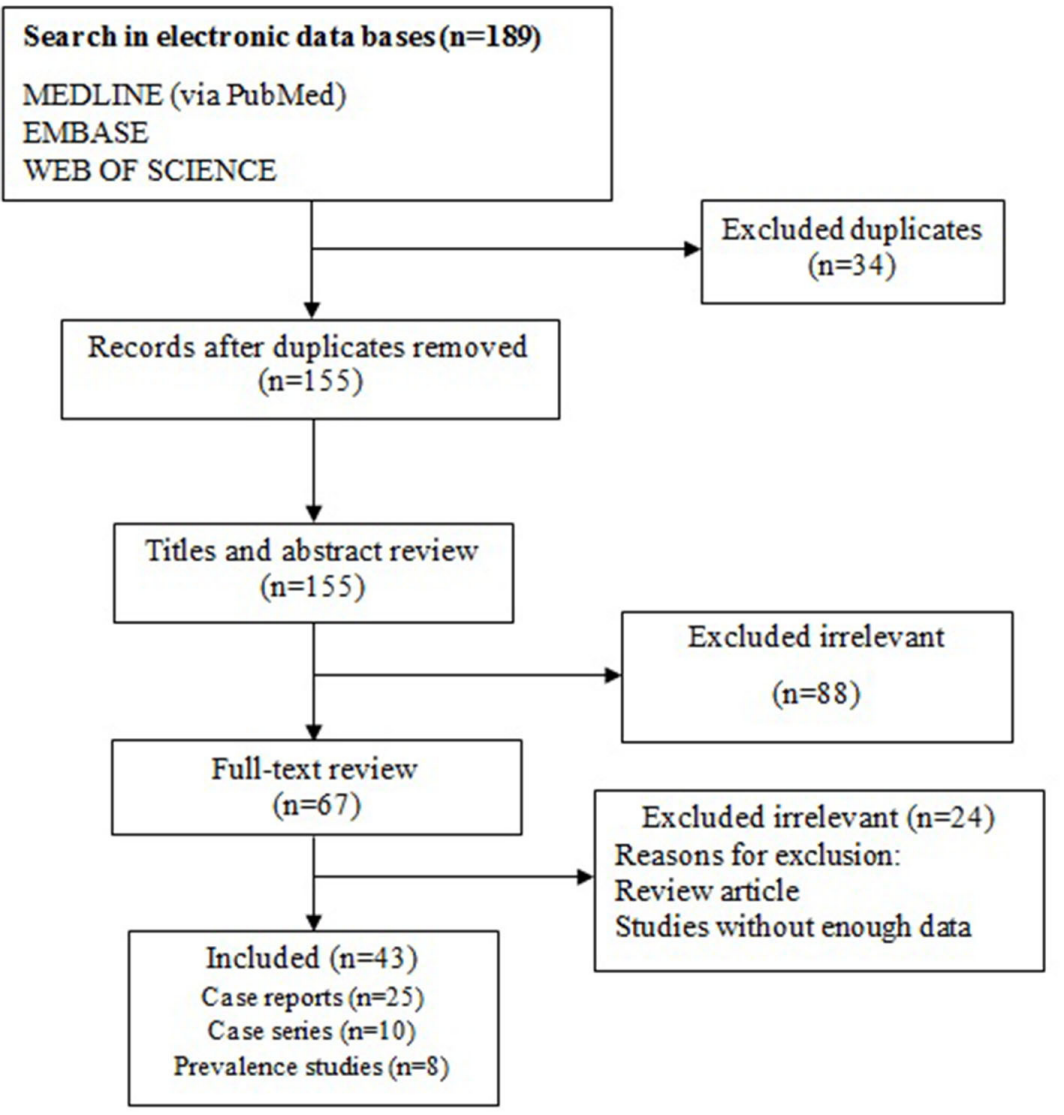
Flow chart of study selection for inclusion in the systematic review.

Overall, 189 citations were recorded in the initial database searches. Our data collection was from three databases and many duplicate studies were included. After removing 34 duplicates, 155 non-duplicate studies remained. Of these, 88 non-relevant studies were removed from our review after checking titles and abstracts. In the step of full-text screening, 24 irrelevant articles were also excluded. Eventually, 43 studies were selected for the final analysis (Figure 1).

### The Frequency of Skin Manifestations Among Patients With COVID-19 Based on Prevalence Studies

**Figure 2:**
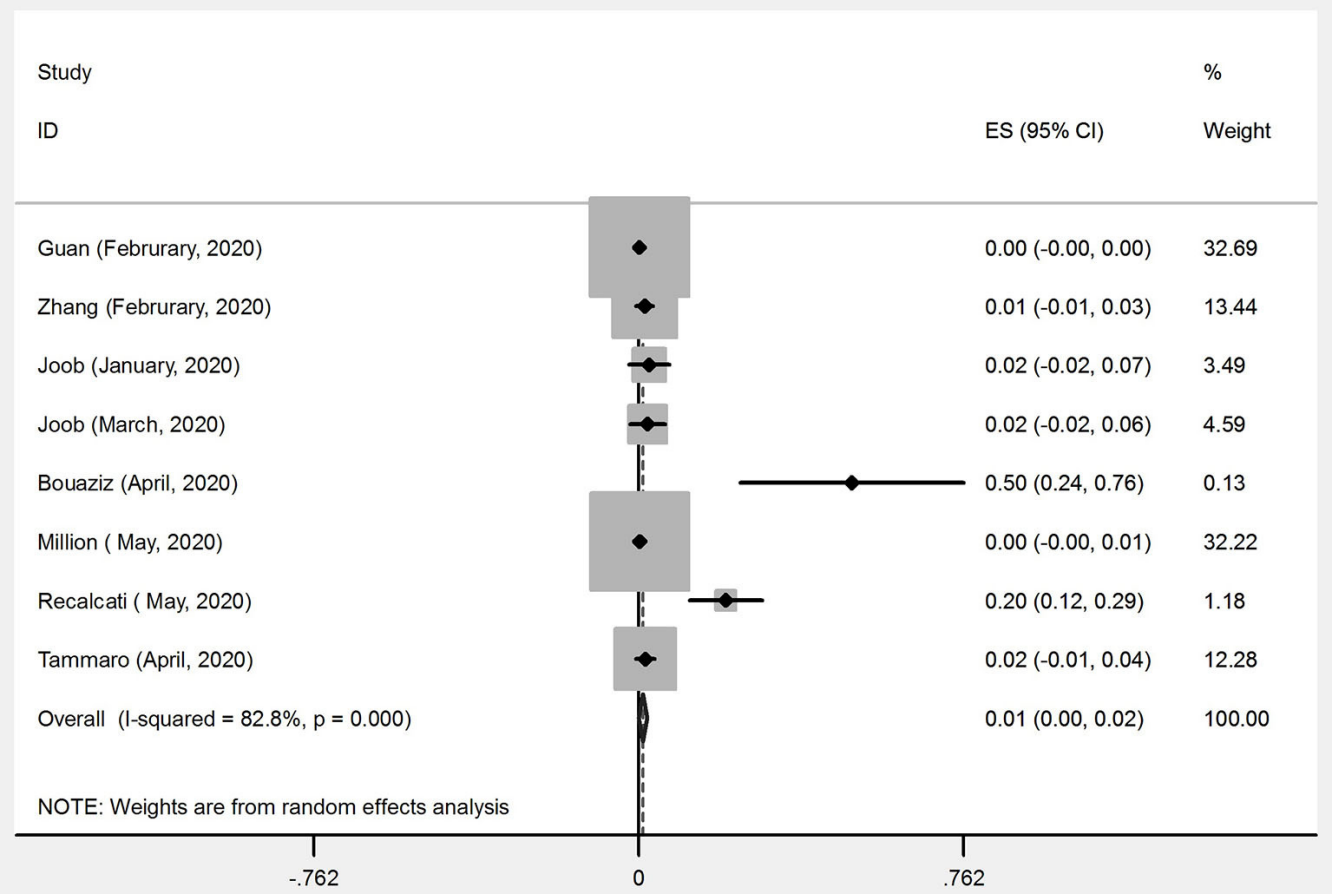
Forest plot of the meta-analysis on the prevalence of skin manifestations among patients with COVID-19.

**Figure 3:**
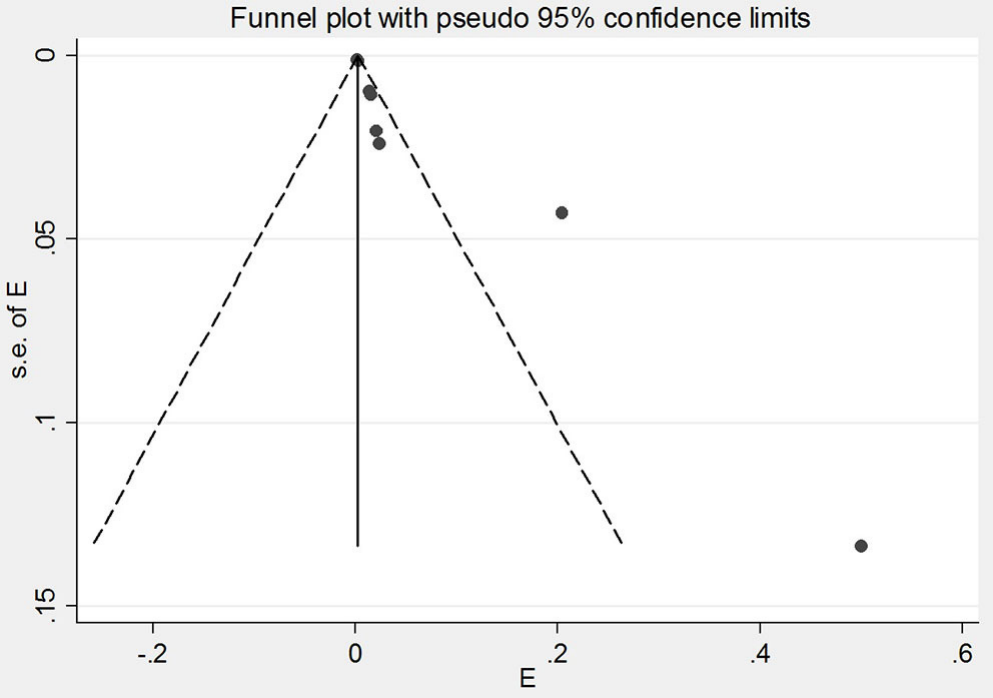
Funnel plot of the meta-analysis on the prevalence of skin manifestations among patients with COVID-19.

**Figure 4:**
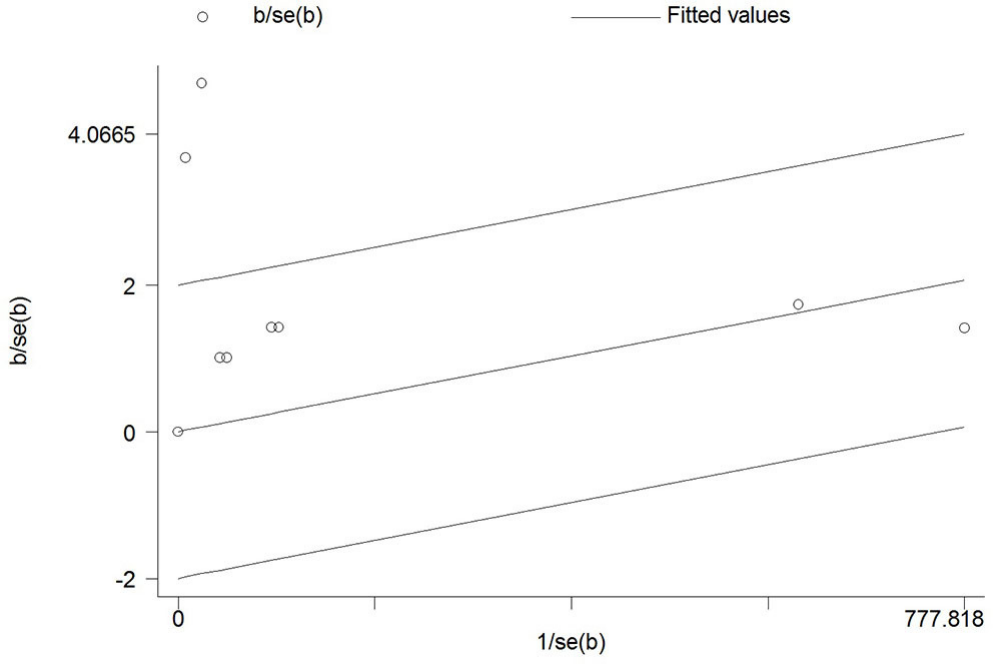
Galbraith of the meta-analysis on the prevalence of skin manifestations among patients with COVID-19.

Out of 43 articles that reported the prevalence of skin manifestations among patients with COVID-19, eight articles (four from Asia and four from Europe) were prevalence studies and 35 articles (six from Asia, 24 from Europe, four from America, and one from Africa) were case reports/case series (Tables 1, 2). A meta-analysis of prevalence studies showed that the frequency of skin manifestations among patients with COVID-19 were reported in four countries (China, Thailand, France, and Italy), with an overall frequency of 1.0% [(95% CI) 0.1–1.9] among 2621 COVID-19 patients (Table 3). Figure 2 is a forest plot, which can summaries almost all of the necessary data of a meta-analysis. The heterogeneity among assessed articles is presented in Figure 3 (called a funnel plot, which is a plot of the intervention effect estimates from individual studies against a measure of study size) and Figure 4 (called a galbraith plot for displaying several estimates of the same quantity that have different standard errors). The numbers and types of skin manifestations among COVID-19 patients were as follow: erythematous rash (15/2,621), urticaria (7/2,621), exanthema (4/2,621), chickenpox like-vesicles (3/2,621), petechia, purpura, chilblain like-lesion and herpetiform lesion (each 2/2,621), and livedo and eruptive cherry angioma (each 1/2,621). Erythematous rash and urticaria were the most skin manifestations among patients with COVID-19.

**Table 1:** Characteristics of included prevalence studies.

**First author**	**Country**	**Published time**	**Type of study**	**Number of patients with COVID-19**	**Number of COVID-19 patients with skin manifestations**	**Mean age**	**Male/** **female**	**Diagnostic method**
Bouaziz (20)	France	Apr-20	Case series	14	7	nr	nr	PCR
Joob (21)	Thailand	Jan-20	Case series	41	1	40.5	4M/37F	real-time PCR
Joob (22)	Thailand	Mar-20	Case series	48	1	nr	nr	RT-PCR
Recalcati (12)	Italy	Mar-20	Case series	88	18	nr	nr	nr
Tammaro (23)	Italy	Apr-20	Case series	130	2	nr	nr	RT-PCR
Zhang (24)	China	Feb-20	Case series	140	2	57	71M/69F	RT-PCR
Million (25)	France	May-20	Case series	1061	3	43.6	492M/569F	PCR
Guan (26)	China	Feb-20	Case series	1099	2	47	639M/460 F	RT-PCR

*RT-PCR, Real time-Polymerase Chain Reaction; nr, not report*.

**Table 2:** Characteristics of Case reports/case series studies.

**First author**	**Country**	**Published time**	**Type of study**	**Number of patients with COVID-19**	**Number of COVID-19 patients with skin manifestations**	**Mean age**	**Male/female**	**Diagnostic method**
Ehsani (27)	Iran	May-20	Case report	1	1	27	M	CT
Ma (28)	China	May-20	Case report	1	1	69	M	IgM/IgG anti-SARS-CoV-2 antibody and CT
Qian (29)	China	Apr-20	Case report	1	1	53	M	RT-PCR
Lu (30)	China	Mar-20	Case report	1	1	nr	nr	RT-PCR
Alramthan (31)	Kuwait	Apr-20	Case series	2	2	31	2F	RT-PCR
Gunawan (32)	Indonesia	May-20	Case report	1	1	51	M	RT-PCR
Henry (33)	France	Apr-20	Case report	1	1	27	F	RT-PCR
Mahé (34)	France	Apr-20	Case report	1	1	64	F	RT-PCR
Zulfiqar (35)	France	Apr-20	Case report	1	1	65	F	RT-PCR
Ahouach (36)	France	Apr-20	Case report	1	1	57	F	Nasopharyngeal swab PCR
Bodard (37)	France	Apr-20	Case report	1	1	45	F	Nasopharyngeal Covid-19 test
Amatore (38)	France	Mar-20	Case report	1	1	39	M	RT-PCR
Sanchez (39)	France	Apr-20	Case report	1	1	nr	F	RT-PCR
Castanedo (40)	Spain	Apr-20	Case report	1	1	61	M	RT-PCR
Moreno (41)	Spain	Apr-20	Case report	1	1	32	F	RT-PCR
Estébanez (42)	Spain	Apr-20	Case report	1	1	28	F	nr
Guimaraens (43)	Spain	Apr-20	Case report	1	1	48	M	Real-time RT-PCR
Landa (15)	Spain	Apr-20	Case series	3	3	42.3	1M/2F	PCR
Olivé (44)	Spain	Apr-20	Case series	2	2	3.1	1M/1F	Nasopharyngeal swab PCR
Test (45)	Italy	May-20	Case report	1	1	70	F	RT-PCR
Mugheddu (46)	Italy	May-20	Case report	1	1	45	M	SARS-CoV-2 swab, chest X-Ray
Sachdeva (17)	Italy	Apr-20	Case series	3	3	73.3	3F	RT-PCR
Locatelli (47)	Italy	May-20	Case report	1	1	16	M	RT-PCR
Zengarini (48)	Italy	May-20	Case report	1	1	67	F	RT-PCR
Paolino (49)	Italy	May-20	Case report	1	1	37	F	Nasopharyngeal and oropharyngeal swab
Genovese (50)	Italy	Apr-20	Case report	1	1	8	F	Nasopharyngeal swab
Gianotti (51)	Italy	Apr-20	Case series	8	2	nr	1M, 1nr	nr
Kolivras (52)	Belgium	Apr-20	Case report	1	1	23	M	RT-PCR
Damme (53)	Belgium	Apr-20	Case series	2	2	55	1M/1F	RT-PCR
Hoehl (54)	Germany	Feb-20	Case series	2	1	nr	nr	RT-PCR
Najarian (55)	America	Apr-20	Case report	1	1	58	M	PCR
Magro (56)	America	Apr-20	Case series	3	3	46	1M/2F	RT-PCR
Manalo (57)	America	May-20	Case series	2	2	57	1M/1F	nr
Hunt (58)	America	Mar-20	Case report	1	1	20	M	Chest radiography, PCR
Janah (59)	Morocco	May-20	Case series	2	2	23	2M	nr

*RT-PCR, Real time-Polymerase Chain Reaction; nr, not report*.

**Table 3:** Frequency of skin manifestations among patients with COVID-19.

**COVID-19 patients with skin manifestations**			**Prevalence%** ** (95% CI)**	**Number of** ** studies**	**Number of** ** patients**	**I-squared**
	Overall		1.0 (0.1–1.9)	8	2,621	82.8%
Subgroup	Asia	China	0.2 (0.0–0.5)	4	1,328	6.4%
		Thailand				
	Europe	Italy	6.6 (1.3–12.0)	4	1,293	91.2%
		France				

### The Frequency of Skin Manifestations Among Patients With COVID-19 Among Different Continents Based on Prevalence Studies

The meta-analysis of prevalence studies showed that the frequency of skin manifestations among patients with COVID-19 was 6.6% (95% CI 1.3–12.0) among 1,293 patients in Europe, and 0.2% (95% CI 0.0–0.5) among 1,328 patients in Asia (Table 3). There has been no report of skin manifestations among COVID-19 patients from America, Africa, and Oceania. As shown in Table 1, the most COVID-19 patients with skin manifestations were reported in Europe (Italy and France with 20 and 10 cases, respectively). Also, the most types of skin manifestations in Italy, France, China, and Thailand were erythematous rash, exanthema, petechiae, and chronic urticaria, respectively.

### The Frequency of Skin Manifestations Among Patients With COVID-19 Based on Case Reports/Case Series

We assessed the skin manifestations among cases with COVID-19 that were reported in the mentioned electronic databases. Characteristics of case reports/case series studies (which were not taken into account during the analyses already mentioned above) are shown in Table 2.

According to the results of the case reports/case series, 30 types of skin manifestations have been reported among patients with COVID-19 from 11 countries (Table 4). Most of the cases were in Italy (11 cases), Spain (nine cases), France (seven cases), and the United States (six cases) (Table 4). Among the patients whose gender was mentioned, 24 patients with skin manifestations were women, and 20 were men. Evaluation of the results of the studied articles demonstrated that out of 54 patients with COVID-19, 48 patients (88.8%) showed skin manifestations. In the field of skin manifestations types, erythematous rash (32 studies), pruritic plaques (13 studies), urticaria (eight studies), and macule (seven studies) were the most reported presentations in included studies (Table 4). Among these, erythematous rash (59.1%) and urticaria (14.8%) were the most common skin manifestations reported in patients with COVID-19.

**Table 4:** Summary of the case reports/case series findings.

**Overall**				
**Types of study**	**Number of studies**	**Total patients with COVID-19**	**Total COVID-19 patients with skin manifestations**	n/N^*^ **(%)**
Case report	25	25	25	25/25 (100.0)
Case series	10	29	23	23/29 (79.3)
	**Variables**	**Number of studies**	**Number of patients with skin manifestations**	n/N^*^ **(%)**
Types of skin manifestations	Eruption	1	1	1/54 (1.8)
	Pustule	1	1	1/54 (1.8)
	Scales	4	4	4/54 (7.4)
	Erythematous	19	19	19/54 (35.1)
	Papule	4	4	4/54 (7.4)
	Plaques	7	7	7/54 (12.9)
	Pruritic	6	6	6/54 (11.1)
	Rash	13	13	13/47 (24.0)
	Petechia	2	2	2/54 (3.7)
	Maculopapula	4	4	4/54 (7.4)
	Macule	7	7	7/54 (12.9)
	Patches	4	4	4/54 (7.4)
	Urticaria	8	9	9/54 (16.6)
	Edematous	2	2	2/54 (3.7)
	Itchy	5	5	5/54 (9.2)
	Lesions	5	8	8/54 (14.8)
	Squamous	1	1	1/54 (1.8)
	Psoriasis	1	1	1/54 (1.8)
	Papulovesicular	2	2	2/54 (3.7)
	Erythematoedematous	1	1	1/54 (1.8)
	Purpura	4	5	5/54 (9.2)
	Exanthema	1	1	1/54 (1.8)
	Livedo	1	1	1/54 (1.8)
	Cyanosis	1	1	1/54 (1.8)
	Hemorrhage	1	1	1/54 (1.8)
	Pain	2	2	2/54 (3.7)
	Ulcer	1	1	1/54 (1.8)
	Papilla	1	1	1/54 (1.8)
	Vasculitis	1	1	1/54 (1.8)
	Gangrene	1	1	1/54 (1.8)
Clinical manifestation	Cough	16	19	19/54 (35.1)
	Diarrhea	6	6	6/54 (11.1)
	Nasal congestion	4	4	4/54 (7.4)
	Fatigue	4	4	4/54 (7.4)
	Myalgia	5	5	5/54 (9.2)
	Arthralgia	4	4	4/54 (7.4)
	Fever	21	27	27/54 (50.0)
	Asthenia	3	3	3/54 (5.5)
	Anorexia	1	1	1/54 (1.8)
	Pain	1	1	1/54 (1.8)
	Headache	6	6	6/54 (11.1)
	Malaise	1	1	1/54 (1.8)
	Sore throat	2	2	2/54 (3.7)
	Dyspnea	2	7	7/54 (12.9)
	Respiratory problems	5	7	7/54 (12.9)
	Rhinorrhea	2	2	2/54 (3.7)
	Gastrointestinal complaints	5	5	5/54 (9.2)
	Dysgeusia	1	1	1/54 (1.8)
	Abdominal discomfort	1	1	1/54 (1.8)
	Weakness	2	2	2/54 (3.7)
	Pyrexia	2	2	2/54 (3.7)
	Hypoxemia	1	1	1/54 (1.8)
	Constipation	1	1	1/54 (1.8)
	Chest pain	3	3	3/54 (5.5)
	Tachycardia	1	1	1/54 (1.8)
	Chills	1	1	1/54 (1.8)
	Anosmia	1	1	1/54 (1.8)
	Ageusia	1	1	1/54 (1.8)
	Insomnia	1	1	1/54 (1.8)
	Transient blurred vision	1	1	1/54 (1.8)
	Confused	1	1	1/54 (1.8)
	Coryza	1	1	1/54 (1.8)
	Lymphonodal enlargement	1	1	1/54 (1.8)
	Pharyngodynia	1	1	1/54 (1.8)
	Pharyngitis	1	1	1/54 (1.8)
Comorbidities	Atopic dermatitis	1	1	1/54 (1.8)
	Chronic urticaria	1	1	1/54 (1.8)
	Diabetes	3	4	4/54 (7.4)
	Hypertension disease	3	3	3/54 (5.5)
	Chronic renal failure	2	2	2/54 (3.7)
	Peripheral artery	1	1	1/54 (1.8)
	Severe psoriasis	1	1	1/54 (1.8)
	Arthritis	1	1	1/54 (1.8)
	Varicella infection	1	1	1/54 (1.8)
	Dyslipidemia	1	1	1/54 (1.8)
	Hyperuricemia	1	1	1/54 (1.8)
	Obesity	4	4	4/54 (7.4)
	High blood pressure	1	1	1/54 (1.8)
	Hypercholesterolemia	1	1	1/54 (1.8)
	nr	26	34	34/54 (62.9)
CT	Ground-glass opacification	7	7	7/54 (12.9)
	bilateral abnormalities	10	12	12/54 (22.2)
	atypical bilateral pneumonia	5	6	6/54 (11.1)
	Normal	3	4	4/54 (7.4)
	nr	15	24	23/54 (44.4)
Laboratory finding	Lymphocytopenia	1	1	1/54 (1.8)
	Leukopenia	1	1	1/54 (1.8)
	Thrombocytopenia	4	4	4/54 (7.4)
	Elevated CRP	6	7	7/54 (12.9)
	Low hemoglobin	1	1	1/54 (1.8)
	Low RBC	1	1	1/54 (1.8)
	Low HCO_3_	1	1	1/54 (1.8)
	Low total CO_2_	1	1	1/54 (1.8)
	High ESR	2	2	2/54 (3.7)
	High WBC	1	1	1/54 (1.8)
	High LDH	1	1	1/54 (1.8)
	Lymphopenia	2	2	2/54 (3.7)
	Increased liver enzymes (GOT, GPT, LDH, GGT doubled)	1	1	1/54 (1.8)
	D dimer elevated	3	4	4/54 (7.4)
	Rising serum creatinines	1	1	1/54 (1.8)
	Elevated INR	1	1	1/54 (1.8)
	nr	20	24	24/54 (54.4)
Isolation source	Nasopharyngeal swab	18	21	21/54 (38.8)
	Nasal swab	1	1	4/54 (7.4)
	Oropharyngeal swabs	4	4	4/54 (7.4)
	Pharyngeal swab	4	4	4/54 (7.4)
	Sputum	1	1	1/54 (1.8)
	nr	14	20	20/54 (37.0)
Outcome	Live	33	44	44/54 (81.4)
	Death	1	1	1/54 (1.8)
	nr	1	2	2/54 (3.7)

* *n, number of patients with any variables; N, the total number of patients with COVID-19; nr, not report*.

The clinical symptoms were also checked in COVID-19 patients with skin manifestations. According to the results of the studies, 35 types of clinical symptoms were reported in these patients (Table 4). Of these, fever (50.0%), cough (35.1%), dyspnea and respiratory problems (12.9% each), and diarrhea and headache (11.1% each) were the most common clinical symptoms among COVID-19 patients with skin manifestations.

Of the 14 types of comorbidities registered in the studies, diabetes (4), obesity and hypertension (three studies for each), and chronic renal failure (2) were the most common comorbidities (Table 4). Accordingly, obesity and diabetes (7.4%) and hypertension (5.5%) were the most common comorbidities reported in patients with COVID-19 with skin manifestations.

In 25 articles, CT scan was mentioned as one of the diagnostic methods used for COVID-19. The results of patients' CT scan were as follows: Ground-glass opacification, bilateral abnormalities, atypical bilateral pneumonia, and normal. Bilateral abnormalities were reported in 10 studies and in 12 (22.2%) patients (Table 4). The results of CTs in most of the evaluated patients were of this type. Laboratory examination of patients showed that elevated C-reactive protein (CRP) (in six studies) and thrombocytopenia (in four studies) were the most common findings (Table 4). These two laboratory findings were in 12.9% (7/54) and 7.4% (4/54) of patients, respectively. The study also looked at the patients' outcomes. According to the analyzed studies, of the 48 patients (mentioned in Table 2), 44 patients returned to life and one died (Table 4). The patient with COVID-19 who died was a 71-year-old man who had acute urticaria. A patient with clinical symptoms (weakness, pyrexia, hypoxemia, pain, constipation, chest pain, and tachycardia) and commodities (type 2 diabetes, hypertension, peripheral artery disease, and chronic renal failure) was reported by Damme in April 2020 (53).

In the case reports/case series studies, the drugs used for treatment of COVID-19 patients with skin manifestations were classified into three sections: antiviral drugs, antibacterial drugs, and a combination of drugs (Table 5). Lopinavir and Ritonavir were the most widely used antiviral drugs (four studies for each) for the treatment of patients. Among the antibacterial drugs listed in Table 5, azithromycin was the most widely used antibacterial agent that was used in six studies. Ceftriaxone and omeprazole were other antibiotics used in two studies to treat patients. Various studies have used different treatment regimens to treat COVID-19 patients. One treatment regimen including Hydroxychloroquine (HCQ) and azithromycin has been used in two studies and other therapies have been reported just in one study. Other drug combinations in addition to antiviral and antibacterial drugs are shown in Table 5 as “others.” As it turns out, HCQ (in 10 studies), Paracetamol (in four studies), and Prednisone (in two studies) were other drug combinations that were used more than others in the treatment of patients.

**Table 5:** Agents used in the treatment of COVID-19 patients.

	**Agent**	**Number of studies**	**n/N**	**%**
Antiviral drug	Lopinavir	4	4	4/54 (7.4)
	Ritonavir	4	4	4/54 (7.4)
	Oseltamivir	1	1	1/54 (1.8)
	Remdesivir	2	2	2/54 (3.7)
	Dihydrocodeine	1	1	1/54 (1.8)
Antibacterial drug	Azithromycin	6	6	6/54 (11.1)
	Cefpodoxime	1	1	1/54 (1.8)
	Ceftriaxon	2	2	2/54 (3.7)
	Cefoperazone-sulbactam	1	1	1/54 (1.8)
	Omeprazole	2	2	2/54 (3.7)
	Amoxicillin–clavulanic acid	1	1	1/54 (1.8)
	Levofloxacin	1	1	1/54 (1.8)
	Piperacillin/tazobactam	1	1	1/54 (1.8)
	Rabeprazole	1	1	1/54 (1.8)
	Metoclopramide	1	1	1/54 (1.8)
	Temozolomide	1	1	1/54 (1.8)
Others	Apremilast	1	1	1/54 (1.8)
	HCQ	10	10	10/54 (18.5)
	Prednisone	2	2	2/54 (3.7)
	Paracetamol	4	5	5/54 (9.2)
	Benzonatate	1	1	1/54 (1.8)
	Heparin	1	1	1/54 (1.8)
Combination therapy	Lopinavir, Ritonavir, HCQ, Oral prednisone	1	1	1/54 (1.8)
	Azithromycin, Benzonatate	1	1	1/54 (1.8)
	Lopinavir, Ritonavir, HCQ, Azithromycin	1	1	1/54 (1.8)
	Temozolomide, Prednisone, Apremilast, Lopinavir, Ritonavir, Ceftriaxon	1	1	1/54 (1.8)
	Azithromycin, HCQ, Cefoperazone-Sulbactam, Omeprazole	1	1	1/54 (1.8)
	Amoxicillin–Clavulanic acid, Heparin	1	1	1/54 (1.8)
	HCQ, azithromycin	2	2	2/54 (3.7)
	HCQ, azithromycin, Remdesivir	1	1	1/54 (1.8)
	Levofloxacin, HCQ	1	1	1/54 (1.8)
	HCQ, omeprazole, Piperacillin, Tazobactam, Remdesevir	1	1	1/54 (1.8)
	Lopinavir, Ritonavir, HCQ, Ceftriaxone, Rabeprazole, Paracetamol, Metoclopramide, Dihydrocodeine	1	1	1/54 (1.8)

*n, number of patients under treatment; N, the total number of patients with COVID-19.

## Discussion

The 2019-nCoV, which causes the COVID-19 pandemic in the world, has infected more than two million people worldwide and caused many deaths in various countries (60). It is therefore a serious threat to human life around the world. Despite valuable and numerous researches having been conducted on this virus, there are still many ambiguities to fully identify its behavior in human pathogenesis. One of the most important issues that can be learned in the future is the association of COVID-19 with other infections. Similar to other RNA viruses, the 2019-nCoV infection can be associated with skin manifestations (14, 38). Recently, evidence of various skin manifestations in patients with COVID-19 has made the disease a major challenge in the world (61, 62). In this regard, the number of reports of skin manifestations in patients with COVID-19 is constantly increasing around the world, especially in European countries (17). In the present study, which is the first systematic review and meta-analysis on COVID-19 and skin manifestations, the prevalence studies as well as case control/case series were evaluated. Accordingly, 2,621 patients with COVID-19 in prevalence studies and 54 similar patients in case control/case series were assessed for skin manifestations. A meta-analysis of prevalence studies showed that most patients with COVID-19 who had skin manifestations were from Europe 6.6% (1.3–12.0) and mainly from Italy and France. Meanwhile, Italy reported the highest number of COVID-19 patients with skin manifestations (20 cases). However, the frequency of these patients was 0.2% (0.0–0.5) in the published studies from Asian countries (China and Thailand). According to the results of the meta-analysis of included studies, the frequency of patients with COVID-19 who also have skin manifestations is higher in Europe than in Asia. Among the reasons that can cause this difference are genetics, age, lifestyle, diet, environmental and geographical parameters of the place that patients live in, health facilities, quality of patient care in hospitals, ability to more accurately diagnose the disease, and other factors. Based on the results of current study, the most common types of skin manifestations were erythematous lesions, rash, and urticaria. On the other hand, evaluation of case reports/case series showed that out of 54 patients with COVID-19 infection, 48 had skin manifestations, most of which were related to European and American countries, respectively. The Asian and African continents ranked next. It is noteworthy to mention that at the time of performing this study there were no reports of skin manifestations in patients with COVID-19 in Oceania. According to data from case reports/case series, the number of female patients was higher than men, and the average age of all patients was 40 years. Analysis of skin manifestations in these studies, similar to those in prevalence studies, showed that erythematous lesion, rash, and urticaria were the most commonly reported skin manifestations among patients with COVID-19. According to research conducted in this field, skin manifestations such as varicella-like exanthem, petechial rash, erythematous rash, and chilblain can occur in the hands, feet, and trunk, and they may sometimes be accompanied by itching and even pain. These skin manifestations can be associated with common symptoms of COVID-19 such as fever, dry cough, and myalgia. Therefore, skin manifestations can be considered as evidence with a diverse range in patients with COVID-19. According to Damme et al. (53) in Belgium, urticaria and pyrexia can be early symptoms of COVID-19, while the patient may not have any respiratory symptoms. Moreno et al. (41) in Spain showed that in a patient with COVID-19 who had fever, myalgia, weakness, cough, and diarrhea, morbilliform itchy rashes appeared on the face, neck, chest, abdomen, and other organs on the sixth day after the onset of main symptoms. The patient had no history of drug use, so skin manifestations could not be linked to drug interactions. Accordant with the results of Recalcati et al. (12) in Italy, in 20.4% of COVID-19 patients, significant skin manifestations occurred. The most common skin manifestations were erythematous rash, urticaria, and vesicle, respectively, which were mainly occurred in the trunk. These people did not have any history of recent drug use leading to drug interactions. According to Guan et al. (26) in China, skin rash was observed in some patients with COVID-19. It should be noted that vesicular rashes are presented in two forms: diffuse and local. The diffuse form is polymorphic, but the local form is monomorphic and only occurs in the trunk. In a study performed by Joob et al. (21) in Thailand, 2.44% of patients developed skin manifestations that were hemorrhagic and petechiae. According to a study by Landa et al. (15) in Spain, patients with COVID-19 showed purpuric manifestations on their fingers. They assume that these skin manifestations could be secondary symptoms of COVID-19. This theory is based on the fact that skin manifestations appear within a few weeks after the peak of infection. Sachdeva et al. (17) in Italy found that 12.5% of people with COVID-19 presented the skin manifestations at the onset of the disease. In another study by Marzano et al. (63), 22 patients with COVID-19 were evaluated. Of these, 16 patients were male. The mean age of patients was 60 years. The average duration of skin manifestations was 8 days. Most clinical manifestations in studied patients were fever, cough, weakness, coryza, dyspnea, hyposmia, hypogeusia, pharyngodynia, diarrhea, and myalgia. The death occurred in three patients. Skin manifestations in these patients were scattered in most cases and were diffuse in 6 patients. In a review by Zhao et al. (64), 44 studies were reviewed, including a total of 507 patients with COVID-19 and skin manifestations. Their results showed that 60.44% of patients were female and the mean age of patients was 49.03 (5–91) years. 488 (96.25%) patients were in Europe. In this study, it was found that skin lesions of patients appeared 1–30 days (mean 9.92) after the onset of systemic symptoms. The maximum incubation period was 30 days and in 13 patients (14.77%), skin lesions were observed as the first symptoms of the disease. Zhao and his colleagues observed that the skin symptoms of the patients were polymorphic, and the most common manifestations were erythema, which was mainly manifested on the trunk. Other lesions were chilblain-like lesions, urticarial-like lesions, vesicular, livedo/necrosis and petechiae, respectively. 44.77% of patients had significant pruritus in skin manifestations. The most common clinical symptoms of studied patients were fever, cough, fatigue, dyspnea, headache, gastrointestinal symptoms, and anosmia, respectively. Laboratory examination of blood samples taken from 39 patients showed that lymphocytopenia, increased CRP and LDH were the most common results, respectively. Out of 507 patients, 13 (2.56%) died. According to a study by Tang et al. (65), of the 16 studies with 256 patients, 88 patients with COVID-19 had skin manifestations (aged between 8–84 years). Skin manifestations were mainly erythematous, urticarial, and vesicular (chicken pox-like or varicelliform). Exanthema was widely presented in the studied patients and was mainly presented on the trunk. Patients often had fever, cough, headache, fatigue, coryza, and dyspnea during the disease. The incubation period of systemic symptoms to exanthema was 2–21 days. In the study by Matar et al. (66), 56 articles including 1,020 patients were reviewed. The results indicated that the rash was the most common skin manifestations in patients with COVID-19. These rashes include erythematous maculopapular/morbilliform, urticarial/annular, vesicular/varicelliform, or petechial/purpuric. Most of the rash manifestations were in the trunk. About 70% of patients had itching. Other symptoms were burning and pain. The average days between the onset of respiratory/systemic symptoms and skin manifestations were ~6.8 days. In some cases, the rash appeared before the systemic symptoms. Therefore, skin manifestations may be considered as a potential factor in the diagnosis and isolation criteria for COVID-19 patients and help to begin the treatment as soon as possible. As stated in this study, the most reported clinical symptoms were fever, cough, dyspepsia, and respiratory problems; and the least ones were anorexia, pain, malaise, dysgeusia, abdominal discomfort, hypoxemia, constipation, tachycardia, chills, anosmia, ageusia, insomnia, transient blurred vision, confusion, and coryza. In a study by Casas et al. (14) in Spain, the clinical manifestations were cough, dyspnea, fever, asthenia, headache, nausea and vomiting, diarrhea, and pneumonia. Another parameter that was evaluated in this study was comorbidities. Based on the results of our study, diabetes, obesity, hypertension, and chronic renal failure were the most common comorbidities in COVID-19 patients with skin manifestations. These underlying diseases were also reported in a study by McMichael et al. (67) in the United States. Among 81 COVID-19 patients admitted to hospital, 69.1% had hypertension, 43.2% had renal disease, and 37.0% had diabetes.

CT scan is one of the diagnostic methods with optimal sensitivity (68). This method can be very helpful in diagnosing COVID-19 patients in order to determine the involvement of the lungs and disease progression. However, this method may not detect lung involvement in the early stages of the disease and may not accurately diagnose the presence of COVID-19 in the patient (69). Based on the results of current study, the findings of CT scan in most COVID-19 patients with skin manifestations have been reported as bilateral patchy shadowing (bilateral interstitial abnormalities and bilateral airspace opacities). However, in some cases, patients had normal CT scans. In other words, skin manifestations appeared in some COVID-19 patients before lung involvement. Other manifestations such as consolidations, thickening of lobar fissures, adjacent pleura, paving patterns, mediastinal lymphadenopathies, and hilar lymphadenopathies were not reported in the patients.

Laboratory findings evaluation revealed that elevated CRP, thrombocytopenia, and increased D-dimer were among the most abnormal laboratory changes. As specified by Zhang et al. (70), this virus increases D-dimer and destroys fibrinogen products leading to acro-ischemia, which is associated with cyanosis of the fingers. Numerous studies have also shown an increase in D-dimer in laboratory findings (71–73). This laboratory evidence is likely to be related to the severity of the disease and skin involvement. However, it is not possible to speak for sure about the exact cause and origin of these laboratory changes in the patients. As mentioned in a study by Zhang et al. (70) in China, an increase in CRP has been reported in 92.14% of 140 COVID-19 patients. In a study by Yang et al. (74) in China, of the 1,476 COVID-19 patients, 20.7% reported thrombocytopenia. Numerous other studies have also reported an increase in thrombocytopenia in COVID-19 patients (75–77). Despite many efforts to control COVID-19, the disease continues to be a major challenge for the global community and a threat to public health. Although much investigate has been made to treat COVID-19 worldwide, there is still no definitive cure. However, supportive therapies such as antipyretics for fever, oxygen therapy for respiratory distress, rest at home, and intake of fluids in the early stages of the disease can be effective (78, 79). In the basis of analysis performed in current study, the most antiviral drugs prescribed to patients included Lopinavir, Ritonavir (as HIV protease inhibitors), and Remdesivir. According to various studies, Lopinavir and Ritonavir have been shown to improve clinical manifestations and reduce the infection rate of viruses (80, 81). Limited clinical information suggest that Remdesivir may have potential activity against COVID-19. Examination of studies showed that in addition to antiviral drugs, azithromycin and HCQ were among the most widely used drugs in the treatment of patients. Different treatment regimens have been used in numerous studies, but only one treatment regimen (azithromycin and HCQ) has been mostly used. Unfortunately, there is not enough information about the effectiveness of the drugs listed in Table 5 and it is not possible to determine the effectiveness of these drugs in treating patients. However, based on the reported results, of the 48 patients in the case reports/case series, only one case resulted in death, which was just treated with HCQ. In general, effective control of infection along with the development of efficient drugs in the treatment of COVID-19 is of great importance for tremendous success in the fight against the disease and reducing mortality. Therefore, it is important to follow infection control protocols in health care. Lack of preventive health measures, insufficient training of employees and lack of infection control programs in the hospitals can play a significant role in increasing the number of COVID-19 patients and possibly in the prevalence of skin diseases and increased mortality rates in these patients. Finally, it is important to mention the limitations of the study. First, in our study, only published articles were reviewed. Second, some of the published reports lacked the inclusion criteria of this study. Third, because there is not enough information in many countries, we were not able to fully demonstrate the prevalence of skin manifestations in COVID-19 patients worldwide. Many COVID-19 patients with skin manifestations may not have been hospitalized and most of them could have been treated at home. This can also affect our inability to fully demonstrate skin manifestations in people with COVID-19 around the world. Fourth, some of the articles did not have sufficient data to analyze the factors mentioned in this study, such as laboratory findings, comorbidities, CT scan results, and the outcome. Fifth: Another limitation of this study was that many articles did not publish sufficient data to assess the side effects of drugs or the side effects of infection. As a result, we have not been able to examine them and discuss in detail the mechanisms of skin manifestations in patients with COVID-19.

## Conclusion

According to the results of included studies, skin manifestations in COVID-19 patients are very diverse and can occur at the beginning of the disease or after antibiotic treatment. Based on our results, skin manifestations including erythematous lesions, rash, and urticaria were the most commonly reported among patients with COVID-19. Identifying these skin manifestations can be a quick way to diagnose some COVID-19 patients. As a result, the importance of this issue is to help stop the spread of COVID-19 and protect other people. In addition to the importance of the side effects of COVID-19, the importance of drug interactions during supportive therapy should also be considered. As a final conclusion, physicians can consider skin manifestations as important clinical features in the diagnosis of patients with COVID-19. However, further study and investigation is needed to confirm and explain an understanding of COVID-19-related skin manifestations.

## Data Availability Statement

All datasets generated for this study are included in the article/supplementary material.

## Author Contributions

MD and FS designed the study. BH and MG conducted the search strategy. FS, SY, PO, MN, and MB performed the data extraction. MD and BH wrote and edited the manuscript. MD carried out the statistical analysis. MD and SS assumed overall responsibility for the accuracy and integrity of the manuscript. All authors contributed to the article and approved the submitted version.

## Conflict of Interest

The authors declare that the research was conducted in the absence of any commercial or financial relationships that could be construed as a potential conflict of interest.
